# An improved window of interest for electroanatomical mapping of atrial tachycardia

**DOI:** 10.1007/s10840-021-00940-0

**Published:** 2021-01-27

**Authors:** Alexis Mechulan, Sok-Sithikun Bun, Alexandre Masse, Angélique Peret, Lauriane Leong-Feng, Frederic Pons, Ahmed Bouharaoua, Pierre Dieuzaide, Sébastien Prévot

**Affiliations:** 1 Ramsay Santé, Hôpital Privé Clairval, Service Cardiologie-Rythmologie, Marseille, France; 2 Departement of Cardiology, Pasteur University Hospital, Nice, France; 3 Biosense Webster France, Johnson & Johnson, Issy les Moulineaux, France; 4 Service de Cardiologie, Hôpital d’Instruction des Armées Sainte-Anne, Boulevard Sainte-Anne, Toulon, France

**Keywords:** Atrial tachycardia, Window of interest, Electroanatomical mapping, Mitral isthmus

## Abstract

**Purpose:**

Diagnosis of atrial tachycardia (AT) with 3D mapping system remains challenging due to fibrosis or previous ablation. This study aims to evaluate a new electroanatomical mapping annotation setting using a window of interest adjusted at the end of the P wave (WOI_p wave_) to identify the AT mechanism more accurately.

**Methods:**

Twenty patients with successful ablation of left AT using navigation system CARTO3 were evaluated. Two maps for each patient were generated offline using either conventional settings of WOI (WOI_conv._) or WOI_p wave_. Three investigators from two centres analysed the maps blindly.

**Results:**

Mechanisms of AT were macroreentrant in 14/20 patients (70%) and focal in 6/20 (30%). WOI_p wave_ resulted in a significant increase in the percentage of correct identification of the mechanism based on mapping alone (93.3 ± 13.7% vs 58.3 ± 33.9%; *p* = 0.0003) compared with WOI_conv._. Diagnoses based on mapping were arrived at faster (27.8 ± 16.4 s vs 38.97 ± 13.64 s, respectively; *p* = 0.0231) and with a greater confidence in the diagnosis (confidence index 2.57 ± 0.45 vs 2.12 ± 0.45, respectively; *p* = 0.0024). With perimitral re-entry specifically “early meets late” was closer to the anatomical region of the mitral isthmus (15.9 ± 20.9 mm vs 48.77 ± 23.23 mm, respectively; *p* = 0.0028).

**Conclusions:**

This study found that electroanatomical mapping acquisition with a window of interest set at the end of the P wave improves the ability to diagnose the arrhythmia mechanism based on the initial map. It is particularly beneficial in identifying area of interest for ablation in perimitral AT.

**Supplementary Information:**

The online version contains supplementary material available at 10.1007/s10840-021-00940-0.

## Introduction

Interpretation of activation mapping to determine the mechanism of atrial tachycardia (AT) may be challenging because of the presence of intrinsic scarring further complicated by previous ablation or fibrosis [1, 2]. With the CARTO3 navigation system, the windows of interest (WOI) is the main parameter used to create an activation map and is defined by the interval preceding and following a reference point that determines the colour of each point projected on the map. WOI onset positioned in mid-diastole has been shown to be effective by De Ponti et al. and is currently the conventional setting (WOI_conv._) [3]. The accuracy of the activation map generated with this technique is crucial for determining the AT propagation. However, apparent circular activation alone may be related to a focus with abnormal conduction through scarred tissue while on the other hand apparent focal activation may be related to failure to appreciate a smaller circuit. Consequently, other settings for WOI have been suggested to better appreciate mechanism and identify a potential critical isthmus [4].

Of course, entrainment and other complementary are critically important diagnostically but are not always possible and AT induction after termination of an AT during entrainment may not be reproducible. In the present study, we aimed to assess whether setting the WOI set at the end of the P wave (WOI_p wave_) improves the ability to diagnose the arrhythmia mechanism based on the initial mapping. In typical atrial flutter (cavotricuspid isthmus-dependent), the plateau phase of slow conduction following the P wave corresponds to the region of a critical isthmus in macroreentrant AT [5]. Positioning the WOI at the end of the P wave prior to a relatively isoelectric component should be related to the entrance into the slow conduction and improve the ability to identify the critical slow conduction zone on the map. Focal AT is generally less problematic, but the P wave onset should reflect anatomical exit from the focus from which it spreads centrifugally [6]. This technique is feasible if there is an identifiable relatively isoelectric interval which there appears to be most of the time.

## Methods

### Study population

The study population included a series of 20 consecutive patients 15% female (mean age: 68.8 ± 9.16 years, range: 48–82 years) undergoing catheter ablation at our institution (Hôpital Privé Clairval, Ramsay Santé, France) for symptomatic sustained left AT. All patients gave their written informed consent before taking part in the study. The study was approved by the local institutional review board (COS-RGDS-2016-06-087-MECHULAN-A) and followed the principles of the Declaration of Helsinki.

### AT ablation procedure

All procedures were performed under general anaesthesia. Using trans-septal access guided by transoesophageal echocardiography, two catheters were inserted into the left atrium via irrigated sheaths (SL0 sheath or Agilis St Jude Medical Inc., St Paul, MN, USA). The mapping catheter used was a duo decapolar electrode catheter (Pentaray®; Biosense Webster Inc., Diamond Bar, CA, USA) to create 3D-electroanatomical mapping of the left atrium (LA) and PVs using a navigation system (Version 4 of CARTO3™; Biosense Webster Inc., Diamond Bar, CA, USA). The second catheter was the contact force (CF) ablation catheter with advanced irrigated porous tip (ThermoCool SmarttouchSF™; Biosense Webster Inc., Diamond Bar CA, USA). 3D-electroanatomical mapping were performed after identification of P waves on the surface electrocardiogram (ECG). The lead V1 was used to identify P waves. When the P wave was not clearly observable, all 12 channels were analysed and ECG signal was amplified. The WOI was set at the end of the P wave and calculated as follows: WOI duration = tachycardia cycle length-10 ms. The WOI started after the P wave when the slope of the signal was equal to zero. Atrial signal on the coronary sinus electrograms was used as the reference signal. A steerable decapolar catheter (Xtrem Dynamic®, Microport CRM France, Clamart), 5 mm electrode spacing, was positioned in the coronary sinus. Points were collected using ConfiDense in wavefront annotation. The tissue proximity indicator (TPI) was activated with LAT filter on 4 ms, stability filter on 4 mm and density filter on 1 mm. The colour fill threshold was set at 5. Signals were colour coded according to their activation timing. After 3D reconstruction, ablations were performed depending on the mechanism identified using the CF catheter with power between 25 and 50 W. Algorithm CARTO3 VISITAG SURPOINT was used to guide the ablation (targeted values: 450 in anterior wall). Mitral line was performed from the superior aspect of the left inferior pulmonary vein to the lateral part of the mitral valve and roof line was done from the left superior pulmonary vein to the right superior pulmonary vein at the top of the posterior wall. The CF used was between 10 and 50 g of force according to the operator’s judgment. Final diagnosis of AT was established by activation map and entrainment and confirmed by the results of catheter ablation achieving AT termination.

### Offline interpretation of map

Maps were generated offline to ensure that all the other parameters remained equal. An engineer used 20 consecutive maps realized in the operating room with the WOI_p wave_. The 20 maps were anonymized and duplicated with the WOI_conv_. All the points were analysed and re-annotated if necessary. The 40 maps were presented randomly to the three investigators from two centres to allow a blind interpretation (Clairval Private Hospital, Marseille and Pasteur University Hospital, Nice). The percentages of correct interpretation of the precise mechanism (defined as correct mechanism and location of AT), time for interpretation (the timer was engaged when the maps were presented to investigators for interpretation) and confidence index for the diagnoses were evaluated. The confidence index was defined as an indicator of the operators’ confidence in their diagnosis: 1, not confident; 2, moderately confident; 3, very confident.

### Measurement of the distance from “early meets late” to the mitral isthmus

Colours on the activation maps were removed and a line was drawn between the inferior side of the left inferior PV and the lateral part of the mitral valve. The distance measured was the smallest distance between “early meets late” and the middle of the mitral isthmus (MI) line [7].

### Statistical analysis

Descriptive variables are presented as means ± standard deviation (SD). Two-group comparisons of continuous variables were performed using Mann-Whitney test as data were not normally distributed according to Shapiro-Wilk tests. Two-tailed *p* values < 0.05 were considered to indicate statistical significance. Statistical analyses were performed using GraphPad Prism (GraphPad software 5.01).

## Results

### Patients and atrial tachycardia characteristics

The study population included a total of 40 maps in 20 patients who presented for first-time AT ablation (*n* = 2) or redo atrial arrhythmia ablation (*n* = 18) because of organized AT. The characteristics of the study population are shown in Table 1. Mean was 68.8 ± 9.16 years and 3 of 20 (15%) were females. Mean LA size was 157.2 ± 34.31 ml.

**Table 1:** Clinical characteristics of the study population

#	Age	Sex	TCL (ms)	LA size (mL)	Mechanism	AT Location	Circuit	Strategy of ablation for macroreentrant tachycardia	Number of previous procedures	Type of index procedure
2	82	F	350	160	Macroreentrant	Perimitral	clockwise	MI Line	1	parox AF
3	63	M	280	230	Macroreentrant	Perimitral	Clockwise	MI Line	Index procedure	–
4	82	M	354	174	Macroreentrant	Perimitral	Clockwise	Anterior Line	1	pers AF
5	65	M	250	91	Macroreentrant	Perimitral	Counterclockwise	MI Line	1	pers AF
7	75	M	240	225	Macroreentrant	Perimitral	Counterclockwise	MI Line	1	AF
8	76	M	290	153	Macroreentrant	Perimitral	Counterclockwise	MI Line	1	pers AF
15	70	M	305	126	Macroreentrant	Perimitral	Clockwise	MI Line	1	pers AF
18	68	M	260	143	Macroreentrant	Perimitral	Clockwise	MI Line	1	parox AF
19	62	M	240	141	Macroreentrant	Perimitral	Clockwise	MI Line	1	pers AF
20	48	M	240	172	Macroreentrant	Perimitral	Clockwise	MI Line	1	pers AF
1	56	M	260	112	Macroreentrant	LA Anterior wall	–	Line from LAA to RPV	1	pers AF
12	78	F	480	150	Macroreentrant	LA Anterior wall	–	Anterior Line	1	parox AF
13	67	M	290	153	Macroreentrant	LA Roof	–	Roof RPV to LPV	1	parox AF
17	64	F	570	178	Macroreentrant	Anterior LAA	–	LAA line	1	pers AF
6	72	M	400	134	Focal	LA Roof	–	–	1	parox AF
9	77	M	270	175	Focal	LA Anterior wall	–	–	1	left AT
10	59	M	240	130	Focal	LA Anterior wall	–	–	1	pers AF
11	76	M	315	150	Focal	LA Roof	–	–	Index procedure	–
14	59	M	360	147	Focal	Ridge	–	–	1	pers AF
16	76	M	330	200	Focal	LA Anterior wall	–	–	1	pers AF

*LA* left atria, *AF* atrial fibrillation, *MI* mitral isthmus, *LAA* left atria appendage, *RPV* right pulmonary vein, *LPV* left pulmonary vein, *TCL* tachycardia cycle length (in ms)

Fourteen of the 20 patients (70%) displayed macroreentrant AT, with perimitral AT the most common arrhythmia occurring in 10 of 14 (71.4%). Six of the 20 patients (30%) displayed focal AT, in the anterior region of the LA (50%), localized in the roof (33.3%), or in the ridge between the LA appendage and left PV (16.7%).

### 3-dimensional electroanatomical mapping

**Fig. 1:**
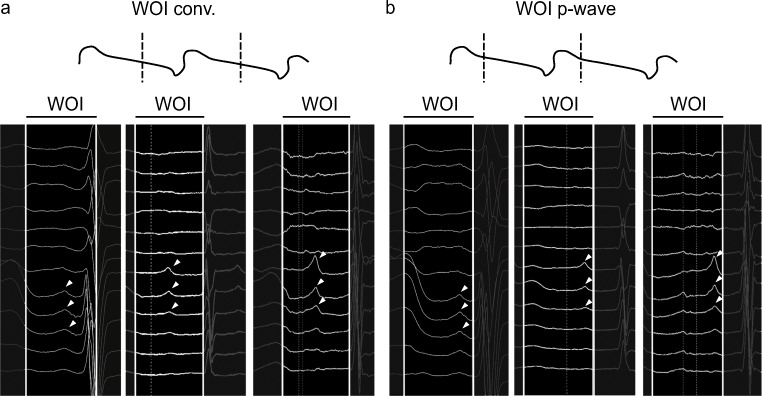
Annotation setting of window of interest on the electroanatomic mapping system interface. **a** Schematic representation of the position of the window of interest (WOI) using the conventional technique (WOI_conv._) on the top. Examples of high magnification of ECG signal allowing clear identification of the P wave (white arrow) using the WOI_conv._ on the bottom. **b** Schematic representation of the position of the WOI set at the end of the P wave (WOI_p-wave_) on the top. Examples of high magnification of ECG signal allowing clear identification of the P wave (white arrow) using the WOI_p-wave_. WOI is the interval between the continuous vertical white line (clear area)

P waves with an isoelectric component were clearly identified on all surface ECGs and WOIs were set. P wave identification was facilitated by clean tracings with patients under anaesthesia. The WOI_conv._ was set using the formula as described by De Ponti et al. [3] with onset of the WOI set in mid-diastole (Fig. 1a). The WOI_p-wave_ was set at the end of the P wave (Fig. 1b). Maps contained an average of 2025.8 ± 978.4 points per map and covered 96.8 ± 1.3% of the AT cycle. Mean cycle length of the clinical ATs was 316.2 ± 86.4 ms (range: 240–570 ms).

**Fig. 2:**
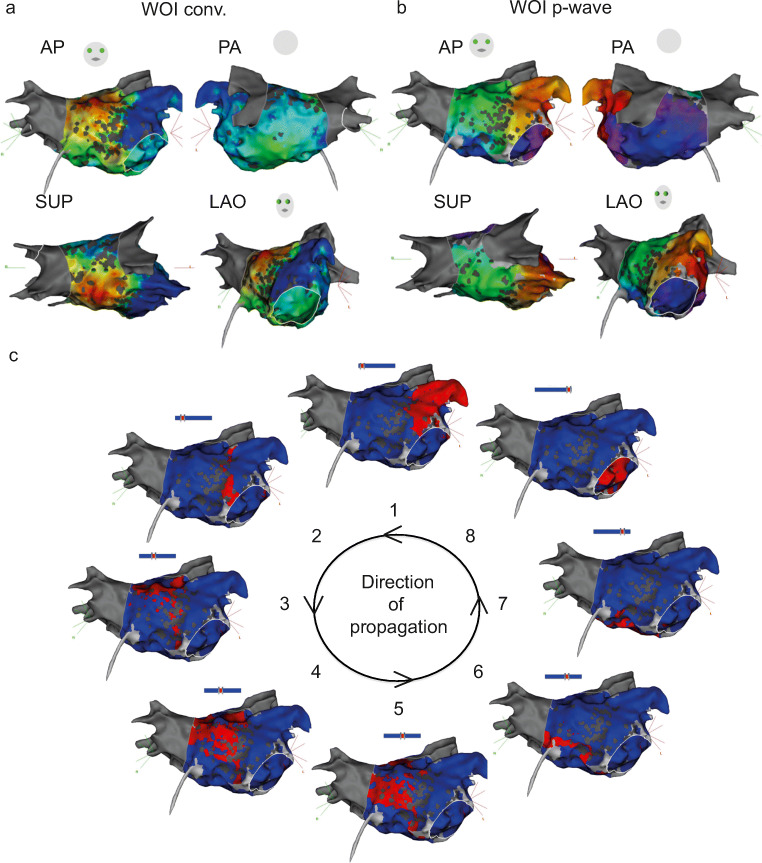
Left atrial 3-dimensional electroanatomical maps. **a** Electroanatomical maps of counterclockwise perimitral AT using the conventional annotation technique (WOI_conv._). **b** Annotation set at the end of the P wave (WOI_p-wave_). **c** Propagation of the AT. *AP* anterior-posterior, *LAO* left anterior oblique, *PA* posterior-anterior, *SUP* superior view

**Fig. 3:**
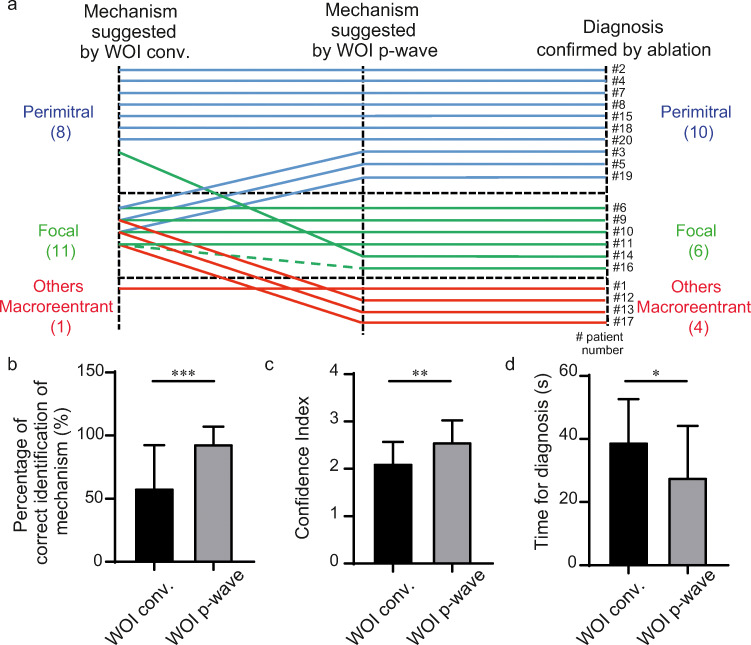
Comparison of the percentage of correct identification of AT using the conventional method and for window of interest (WOI) set at the end of the P wave. **a** Example of diagnostic flowchart for one investigator. The vertical left dotted line represents the mechanism of AT suggested by the conventional WOI (WOI_conv._), the middle one represents the investigational annotation (WOI_p-wave_) and the right one represents the validated diagnosis confirmed by ablation. Each horizontal line symbolizes one patient and the colours indicate the AT mechanism (blue for perimitral, green for focal and red for other macroreentrant ATs). Note that the green dotted line indicates an error of focal location. **b** Percentage of correct identification calculated for all operators; ***p = 0.0003. **c** Confidence in diagnosis; **p = 0.0024. D. Time for diagnosis; *p = 0.0231. In this and the following figure: error bars = SD

Generation of complete maps was achieved in all 20 ATs using the WOI_conv._ (Fig. 2a) or the WOI_p-wave_ (Fig. 2b). The validated diagnosis of AT was defined by comprehensive mapping with clear propagation (Fig. 2c and Supplementary videos 1 and 2) and confirmed by the results of catheter ablation terminating AT. An example of one investigator’s diagnostic flowchart is shown in Fig. 3a. The percentage of correct identification of the mechanism was statistically higher with the WOI_p-wave_ compared with the WOI_conv._ (93.3 ± 13.7% vs 58.3 ± 33.9%, respectively; *p* = 0.0003) (Fig. 3b). A superior degree of confidence in the diagnoses was obtained with the WOI_p-wave_ (confidence index 2.57 ± 0.46 vs 2.12 ± 0.45, respectively; *p* = 0.0024) (Fig. 3c). Time needed for correct identification of the AT mechanism was lower with the WOI_p-wave_ (27.8 ± 16.36 s vs 38.97 ± 13.65 s, respectively; *p* = 0.0231) (Fig. 3d). The inter-observer variability is shown in Table 2.

**Table 2:** Inter-observer variability

	Operator 1				Operator 2				Operator 3			
	WOI Conv.		WOI p-wave		WOI Conv.		WOI p-Wave		WOI Conv.		WOI p-wave	
	Mean	SD	Mean	SD	Mean	SD	Mean	SD	Mean	SD	Mean	SD
Success (%)	65	48.94	85	36.63	65.0	48.9	100	0.0	45	51.04	95	22.36
Index	2.25	0.72	2.55	0.76	1.9	0.8	2.5	0.8	2.25	0.55	2.65	0.59
Time (s)	40.9	26.82	22.45	17.37	52.6	38.07	44.4	39.41	23.4	12.78	16.6	10.83

### Perimitral atrial tachycardias

**Fig. 4:**
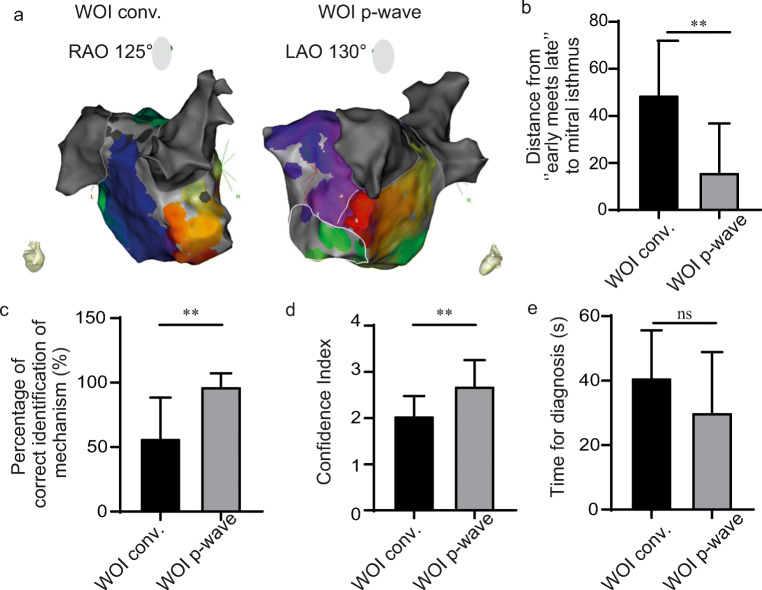
“Early meets late” location for perimitral AT. **a** Electroanatomical map of perimitral AT using the conventional annotation technique (WOI_conv._) (left) or window of interest set at the end of the P wave (WOI_p-wave_) (right). The white line indicates the theoretical location of the mitral isthmus. **b** Distance from “early meets late” to the mitral isthmus in both configurations; **p = 0.0028. **c** Percentage of correct identification calculated for all investigators; **p = 0.0031. **d** Confidence in diagnosis **p = 0.005. **e** Time for diagnosis; ns, *p* = 0.1903. *LAO* left anterior oblique, *RAO* right anterior oblique

Using WOI_p-wave_, 90% of perimitral AT maps clearly showed an “early meets late” located close to the anatomical region of MI (Fig. 4a). This observation was quantified by blindly measuring the distance from “early meets late” to the anatomical MI in both settings. In maps obtained with the WOI_conv._, the distance of “early meets late” from the anatomical MI was higher compared with WOI_p wave_ (48.8 ± 23.2 mm vs 15.9 ± 20.9 mm, respectively; *p* = 0.0028) (Fig. 4b). These results were associated with an increase in the percentage of successful identification of the mechanism with the WOI_p wave_ (96.7 ± 10.5% vs 56.7 ± 31.6% with WOI_conv._) (Fig. 4c) and with an increase in the confidence index (2.7 ± 0.55 vs 2.03 ± 0.43 with WOI_conv._) (Fig. 4d). Time to obtain the diagnosis was not statistically different between the two configurations (30.0 ± 18.9 s vs 40.8 ± 14.9 s; *p* = 0.1903) (Fig. 4e).

### Focal atrial tachycardias

**Fig. 5:**
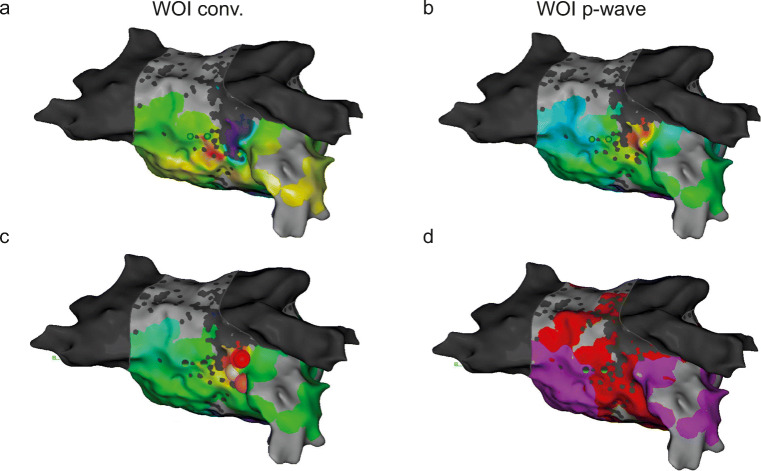
Focal atrial tachycardias. **a** Electroanatomical map of focal AT using the conventional annotation technique (WOI_conv._). **b** Electroanatomical map of focal AT using the investigational annotation technique (WOI_p-wave_). **c** Electroanatomical map showing ablation site indicated by red dots perfectly correlated with earliest point on WOI_p-wave_. **d** Voltage map showing low voltage in red explaining a slow conduction area. All maps are in superior view

Only 6 patients had focal AT and there was no difference between the WOI_conv._ and WOI_p-wave_. These results could be explained by the low number of cases. Generally, the p-wave onset should reflect anatomical exit from the focus. However it is not possible to distinguish micro-reentry from true focal activity with activation mapping. Interestingly, in one case, the origin of AT was colour coded in purple at the end of WOI_conv._ (Fig. 5a) and in red at the beginning of WOI_p-wave_ (Fig. 5b). The origin of AT is separated by 10.4 mm between the two WOIs. This suggests that WOI_p-wave_ increase the sensitivity to detect the first focal activities that could emerge from very slow conduction areas due to scar (Fig. 5 c–d).

## Discussion

This study showed that WOI_p-wave_ facilitated identifying the critical isthmus and the mechanism of AT with the initial maps with CARTO3 prior to further manoeuvres.

Wave-front propagation using activation mapping of scar-related AT can be difficult to interpret. A focus may exhibit apparent circular activation and early meets late even in re-entrant circuits is influenced by the WOI. It is desirable to get as much information from the mapping prior to manoeuvres since the tachycardia may be terminated or accelerated by the latter and be difficult to reinduce. The realization of a high definition activation map is the first essential step in the diagnosis. Other tools are also available to operators such as voltage maps and propagation maps to understand AT mechanism. Even if those tools are very important, they can be used only after completion of the map. On the contrary, the activation map built in real time may allow physician to focus on critical areas. The WOI setting is mandatory to create an activation map, and its modulation only induces a phase-shift of the activation pattern without affecting the sequence of activation [8, 9]. The conventional setting of WOI has been described by De Ponti et al. using a specific formula including the length of the tachycardia cycle, the duration of the P wave and the interval between the P wave and the reference signal. In this study, we evaluated a different approach for the setting of the WOI that allowed the WOI to reflect the transition into the critical slow conduction zone in macroreentrant circuits. In macroreentrant tachycardia, as in typical atrial flutter (cavotricuspid isthmus-dependent), the plateau phase following the P wave corresponds to the region of slow conduction where the cavotricuspid isthmus is located [5]. This proved to be applicable to other macrorentrant tachycardias in our study. WOI_p-wave_ allowed accurate identification of the mechanism on the basis of the initial maps in 93% of cases compared with 58% with the WOI_conv._. Diagnoses were more consistent between all blinded investigators explaining these results. These can be explained by a reduction in misinterpretation of perimitral macroreentrant AT as focal tachycardia. Investigators confused more perimitral macroreentrant ATs with the WOI_conv._ (43.3%). Perimitral were misinterpreted as being focal in 53.8% (*n* = 7) of maps, as macroreentry of the roof in 38.5% (*n* = 5) and as macroreentry of the anterior wall of the LA in 7.7% (*n* = 1). This observation is illustrated in the flowchart in Fig. 3a where 30% of perimitral ATs and 75% of other macroreentrant circuits were misinterpreted as focal ATs by one investigator.

Our study shows that the choice of projected colours plays an important role in the diagnostic accuracy. The colour interpolation feature of CARTO3 software and the physiology of the human eye regarding colour perception could be factors. First, scar due to previous linear lesions or ablation of fractionated signals induces complex patterns of propagation that can result in low-density mapping especially within and around scars. Limited point density in conduction block areas when it corresponds to “early meets late” location, allows over-interpolation within large areas. This could give the appearance of focal AT with centrifugal activation, masking a macroreentrant mechanism [10]. An example of this phenomenon is showed in Fig. 2; scars from the anterior region of the LA force the activation wave to propagate through a small channel. The insufficient number of points close to the scar will lead the system to interpolate colours that do not correspond to real measured points inducing investigators to misinterpret the tachycardia as focal. Biosense with an upgraded HD colouring module realized after our study has corrected this CARTO3 feature. Secondly, we observed that in 90% of maps, “early meets late” was located in the anatomical MI with the WOI_p-wave_ (only one was not located close to the anatomical MI). This observation leads the software to colour wider areas in green. As the perception of eyes is most sensitive to green light [11] (located in the middle of the visible spectrum), eyes identify a large proportion of green in the isochronal colour scale. However, discrimination of fine contrast in shades of green appears to be more difficult and prevents operators from seeing details in those areas. Since the purple/red area was mainly located in the MI, this could explain why all investigators gave a correct interpretation of perimitral AT. Perimitral AT could be terminated with several methods; in our study the ablation line was performed in the MI. The WOI_p-wave_ forces the colour distribution of the map in the region of the isthmus into the red and purple regions allowing better discrimination of the range of activation.

This study has limitations. Evaluation in a larger number of patients with a wider range of complex ATs and real-time assessment with upgraded version of CARTO software is required to further evaluate the clinical usefulness of our WOI. Finally, in the present study, no difference was made between micro-reentry and focal.

## Conclusion

In conclusion, when compared retrospectively with conventional WOI settings, our novel WOI based on end of P wave performed better with respect to the investigator’s percentage of correct identification, confidence index and time of diagnosis. It has particular value for identifying perimitral AT without affecting the identification of other tachycardias.

## Supplementary Information

Supplementary Video 1 AT propagation map shown in fig. 2 using the conventional window of interest. (MP4 6501 kb)

Supplementary Video 2 AT propagation map shown in fig. 2 using the window of interest set at the end of the P wave. (MP4 6794 kb)

## Data Availability

The data will not be deposited but are available on request.
